# Phytic Acid Demonstrates Rapid Antibiofilm Activity and Inhibits Biofilm Formation When Used as a Surface Conditioning Agent

**DOI:** 10.1128/spectrum.00267-23

**Published:** 2023-05-16

**Authors:** Rania Nassar, Mohannad Nassar, Abiola Senok, David Williams

**Affiliations:** a College of Medicine, Mohammed Bin Rashid University of Medicine and Health Sciences, Dubai, United Arab Emirates; b School of Dentistry, College of Biomedical and Life Sciences, Cardiff University, Cardiff, United Kingdom; c Department of Preventive and Restorative Dentistry, College of Dental Medicine, University of Sharjah, Sharjah, United Arab Emirates; The Ohio State University Division of Biosciences

**Keywords:** *Candida albicans*, *Enterococcus* faecalis, phytic acid, antibiofilm, biofilms, endodontic infections, natural antimicrobial products, surface conditioning agent

## Abstract

Root canal infections are associated with biofilms and are treated with chemical irrigants with a high success rate. However, treatment failure does arise, which is attributed primarily to resistance exhibited by biofilms. Currently used irrigants in root canal treatment have disadvantages, and there is therefore a need for more biocompatible alternatives with antibiofilm properties to reduce root canal treatment failure and complications. The aim of this study was to evaluate the *in vitro* antibiofilm properties of phytic acid (IP6), which is a potential alternative treatment agent. Single- and dual-species biofilms of Enterococcus faecalis and Candida albicans were developed on the well surfaces of 12-well plates and on hydroxyapatite (HA) coupons, and then exposed to IP6. In addition, selected HA coupons were preconditioned with IP6 before biofilm development. IP6 demonstrated bactericidal effects and altered the metabolic activity of biofilm cells. Confocal laser-scanning microscopy showed that IP6 caused significant and rapid reduction in live biofilm cells. At sublethal concentrations, IP6 did not alter the expression of tested virulence genes except for C. albicans *hwp1*, the expression of which was upregulated but not reflected by a change in hyphal transformation. IP6-preconditioned HA coupons led to extensive inhibition of dual-species biofilm formation. The results of this study highlight for the first time the antibiofilm inhibitory properties of IP6 and the potential for its exploitation in several clinical applications.

**IMPORTANCE** Root canal infections are biofilm associated, and despite mechanical and chemical treatment procedures, infection recurrence occurs, and this is likely due to the high tolerance of associated biofilms to antimicrobials. The currently used treatment agents have several disadvantages, which necessitates the search for new improved agents. In this study, the natural chemical phytic acid was found to exhibit antibiofilm activity against established mono and dual mature biofilms over a short contact time. Most importantly, phytic acid was found to cause significant inhibition of dual-species biofilm formation when used as a surface preconditioning agent. The findings of this study identified a novel use of phytic acid as a potential antibiofilm agent that can be used in several clinical applications.

## INTRODUCTION

Biofilms are microbial lifestyles that occur on biotic and abiotic surfaces in different settings (1). Eighty percent of human infections are estimated to have a biofilm origin (2). Biofilm microorganisms can be up to 1,000-fold more tolerant to certain antimicrobials (3), and the infections they cause are therefore often persistent and recurrent and present significant treatment challenges (4). Oral infections in humans, including those of the root canal, are typical biofilm-associated infections. Both Enterococcus faecalis and Candida albicans are frequently recovered from persistent infections where endodontic treatment has failed (5–7). Enterococcus faecalis can grow as a biofilm on root canal walls in monotypic infections without synergistic support from other bacteria (8, 9). Candida albicans is primarily co-isolated with other bacteria, especially E. faecalis (10, 11), from persistent infections in the root canal, and this could be attributed to synergistic interactions between these two species.

The mainstay of treatment for endodontic infections involves mechanical debridement and chemical disinfection for biofilm elimination (12). Ethylenediaminetetraacetic acid (EDTA) and sodium hypochlorite (NaOCl) are the most popular irrigants used for this purpose (13). At concentrations between 2.50 and 5.25%, NaOCl is the most frequently used disinfectant (14) in endodontic treatment despite the risk it presents to tooth integrity, the surrounding tissues, and patient safety (15). Studies have shown that biofilm disinfection is significantly improved when using higher volumes and concentrations of NaOCl with an extended application time (16). However, this can negatively affect dentine strength and increases the likelihood of tooth fracture and loss. The antimicrobial effect of NaOCl is also reduced in the presence of organic and inorganic matter (17–19). NaOCl cannot remove the organic smear layer formed during mechanical debridement (20), and, thus, a chelating agent is required. Proposed benefits of smear layer removal include better disinfection and penetration of endodontic sealers into the dental tubules (21, 22). Bacteria that reside within the smear layer, if not eliminated, can recolonize and cause recurrent infection. Hence, it would be potentially beneficial to use a chelating agent that has dual activity, namely, an ability to remove the smear layer and having antimicrobial activity. Importantly, use of a chelating agent with antimicrobial function would contribute to the disinfection process and help reduce the required NaOCl concentration and exposure time. EDTA (15 to 18%) is the most commonly used chelating irrigant (14) but has several drawbacks, including host cell toxicity toward periapical tissue and a lack of antimicrobial activity, which have a negative impact on treatment outcome (23–25). EDTA is also not readily biodegradable, and concerns exist about extrusion into the periapical tissue (26–28). Therefore, given the recalcitrant nature of biofilms, identification of novel chelating agents with antimicrobial and antibiofilm proprieties is required to aid in the achievement of favorable treatment outcome.

Phytic acid (IP6) is a natural agent that has been proposed as a potential root canal irrigant because of its ability to remove the smear layer and its higher biocompatibility than EDTA (29). In general, the antimicrobial effect of IP6 has not been studied in the context of dentistry and specifically in endodontics. Limited research has investigated IP6 on foodborne microorganisms. Kim and Rhee studied its effect on Escherichia coli, and the proposed antimicrobial mechanism was by cell membrane disruption (30, 31). Zhou et al. also found that IP6 was effective against E. coli, Staphylococcus aureus, Bacillus subtilis, and Salmonella enterica serovar Typhimurium (32). In a recent study, IP6 showed inhibition of Clostridium perfringens spore germination and vegetative cell growth (33). However, despite the potential antimicrobial activity of IP6 found in these previous studies, this activity was mainly investigated against planktonic cultures of food-associated pathogens. We have previously reported the broad-spectrum antibacterial activity of IP6 using planktonic cultures and antibiofilm effects on early single-species biofilms (34). However, because biofilm tolerance to antimicrobial agents is dependent on biofilm maturation, microbial composition, and growth conditions, it is important to investigate the effect of IP6 on mature biofilms under conditions that better mirror those of endodontic infections. The aim of this study was therefore to evaluate antibiofilm effectiveness of IP6 and the associated mechanisms against mature biofilms on clinically relevant surfaces. Both static and dynamic biofilm models were used to achieve the intended aims of this research.

## RESULTS

### Antibiofilm activity of IP6 on single- and dual-species biofilms developed using a static model.

**FIG 1:**
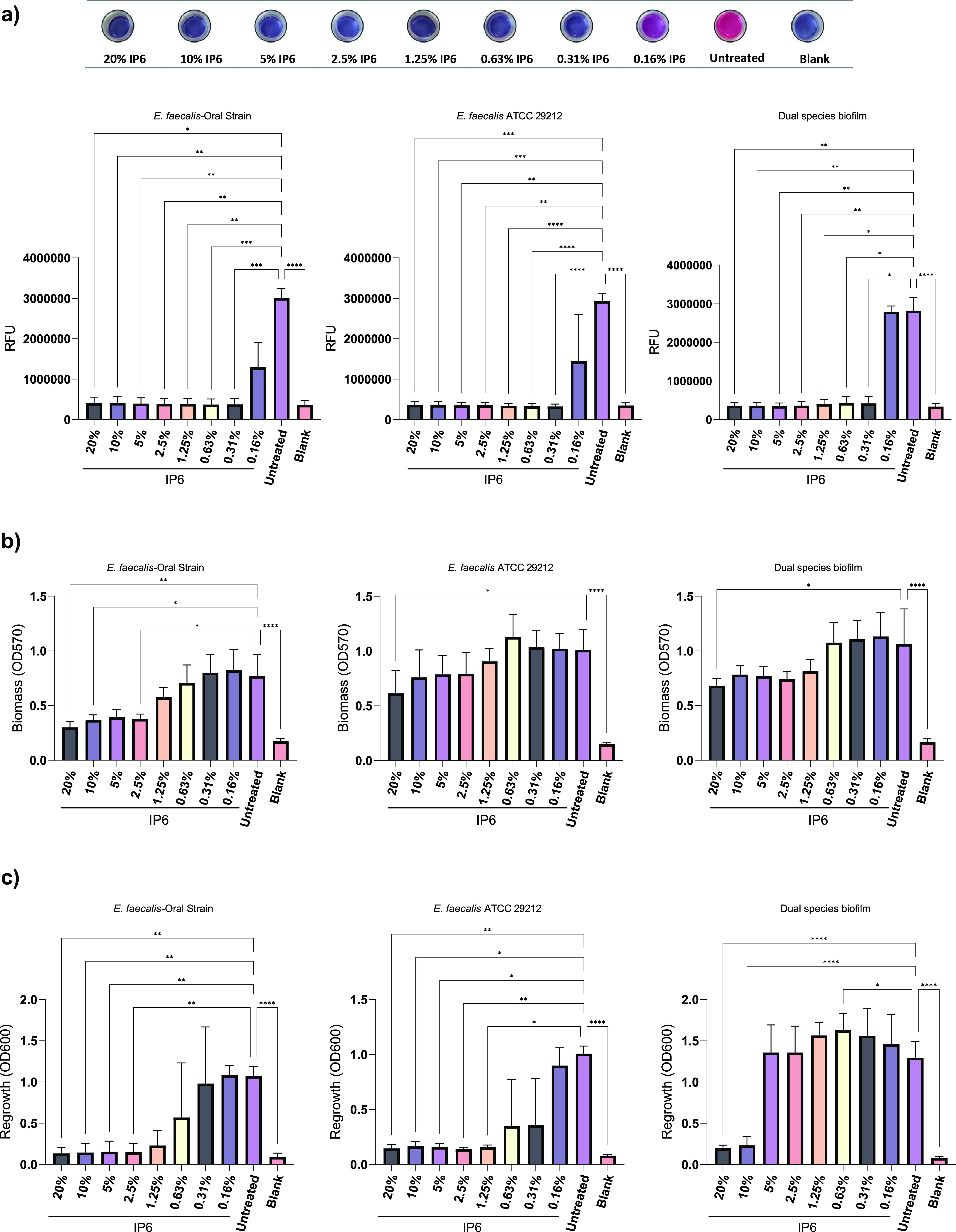
Effect of IP6 on metabolic activity (a), biomass (b), and complete eradication (c) of mono- and dual-species biofilms developed in the wells of a 12-well plate and treated for 24 h. Data are expressed as means of at least three independent experiments, each including at least two replicates. Error bars represent standard deviation (SD); *, *P* < 0.05; **, *P* < 0.01; ***, *P* < 0.001; ****, *P* < 0.0001.

**FIG 2:**
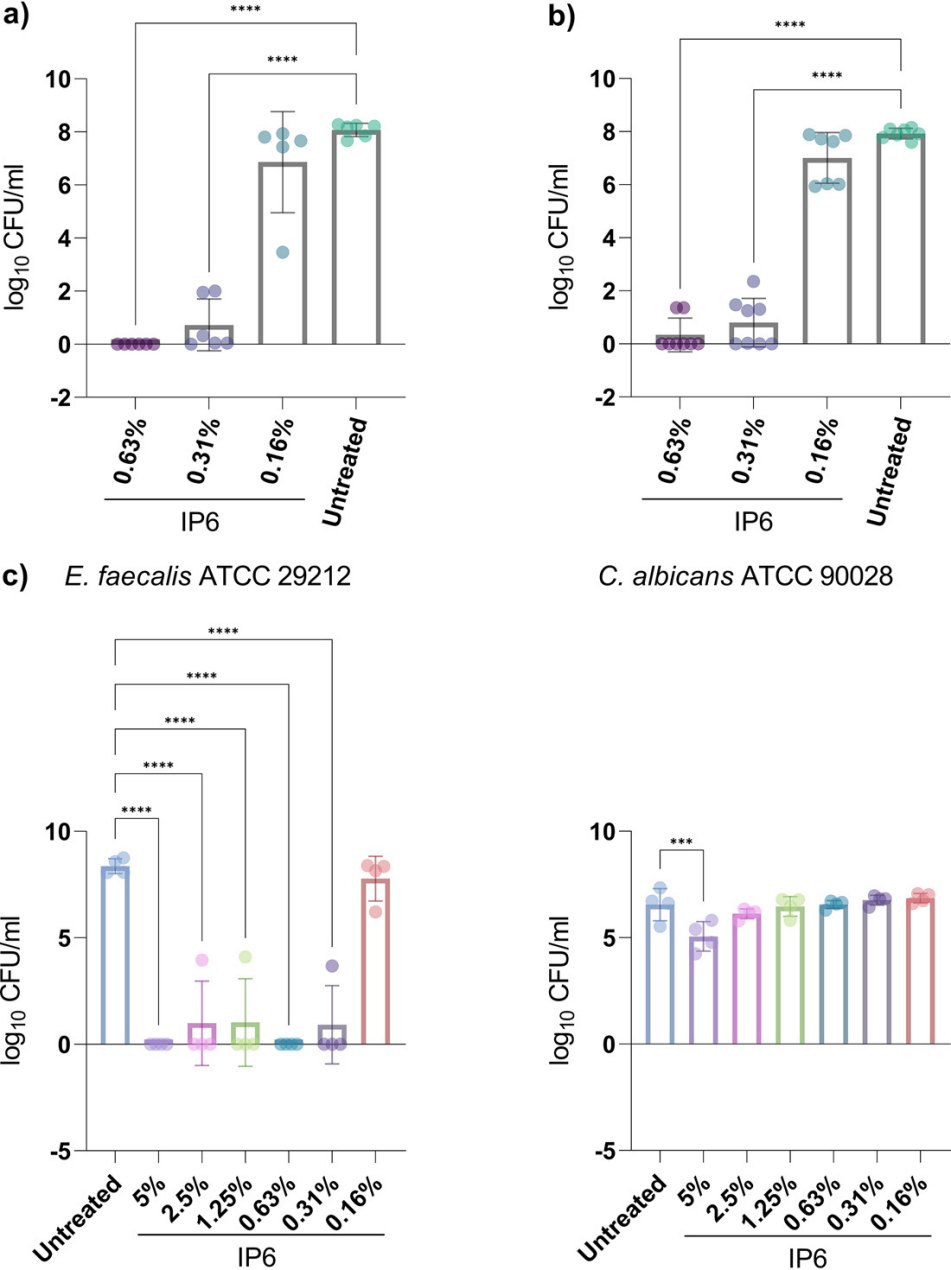
Effect of IP6 on recovered CFU/mL from 24-h mono-species biofilms of E. faecalis oral strain (a) and E. faecalis ATCC 29212 (b) and dual-species biofilm of E. faecalis ATCC 29212 and C. albicans ATCC 90028 (c) developed in the wells of a 12-well plate and treated for 24 h. Data are expressed as means of at least three independent experiments, each including at least one replicate. Error bars represent SD; ***, *P* < 0.001; ****, *P* < 0.0001.

The metabolic activity and total biomass of biofilms after treatment with different concentrations of IP6 were determined using alamarBlue and crystal violet (CV) assays, respectively. The means of relative fluorescence units (RFU; metabolic activity) and absorbance (optical density at 570 nm [OD_570_]; biomass) were calculated. AlamarBlue is a redox indicator, and metabolically active cells reduce the blue color resazurin to resorufin, which has a fluorescent pink color. The inability of cells to reduce alamarBlue is indicative of reduced metabolic activity. Generally, a significant reduction in mean RFU in treated groups compared to untreated groups occurred (Fig. 1a). The exception was when single-species E. faecalis biofilms and dual-species biofilms of E. faecalis and C. albicans were treated with 0.16% IP6. Only the highest concentration of IP6 (20%) significantly reduced the biomass of single-species E. faecalis ATCC 29212 (*P* = 0.017) biofilms and dual-species biofilms (*P* = 0.024). For E. faecalis (oral strain) biofilms, significant biomass reduction was seen at lower IP6 concentrations (20%, 10%, and 2.5%) (Fig. 1b). After challenging biofilms with IP6, the ability of cells to regrow after treatment was assessed. The assay revealed that the lowest IP6 concentrations with 100% bactericidal effect (prevented regrowth) on E. faecalis ATCC 29212 and the E. faecalis oral strain were 1.25% and 2.5%, respectively. For dual-species biofilms, IP6 at 10% and 20% prevented microbial regrowth (Fig. 1c). Since regrowth assessment usually underestimates antimicrobial activity, the microbial count reduction after treatment with IP6 concentrations deemed unsuccessful in completely eradicating the biofilm (in the regrowth assessment) was assessed. Biofilm treatment with IP6 (0.31% and 0.63%) led to a significant log reduction in recovered CFU for single-species biofilms (Fig. 2a and b). For dual-species biofilms, a significant reduction was only evident for E. faecalis ATCC 29212 (Fig. 2c).

### Antibiofilm activity of IP6 on mono- and dual-species biofilms developed on a hydroxyapatite (HA) coupon.

**FIG 3:**
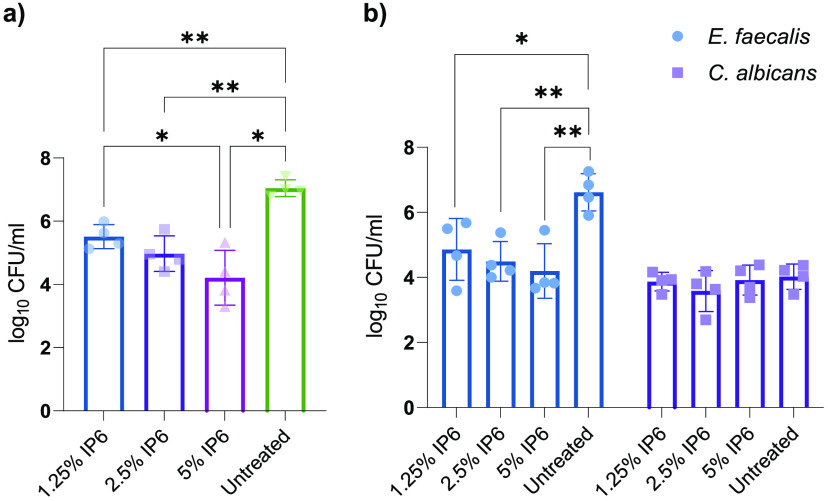
Effect of 5-min IP6 treatment on recovered CFU/mL of mono-species biofilm of E. faecalis ATCC 29212 (a) and dual-species biofilm of E. faecalis ATCC 29212 and C. albicans ATCC 90028 (b) developed on HA coupons. Data are expressed as means of four independent experiments. Error bars represent SD; *, *P* < 0.05; **, *P* < 0.01.

**FIG 4:**
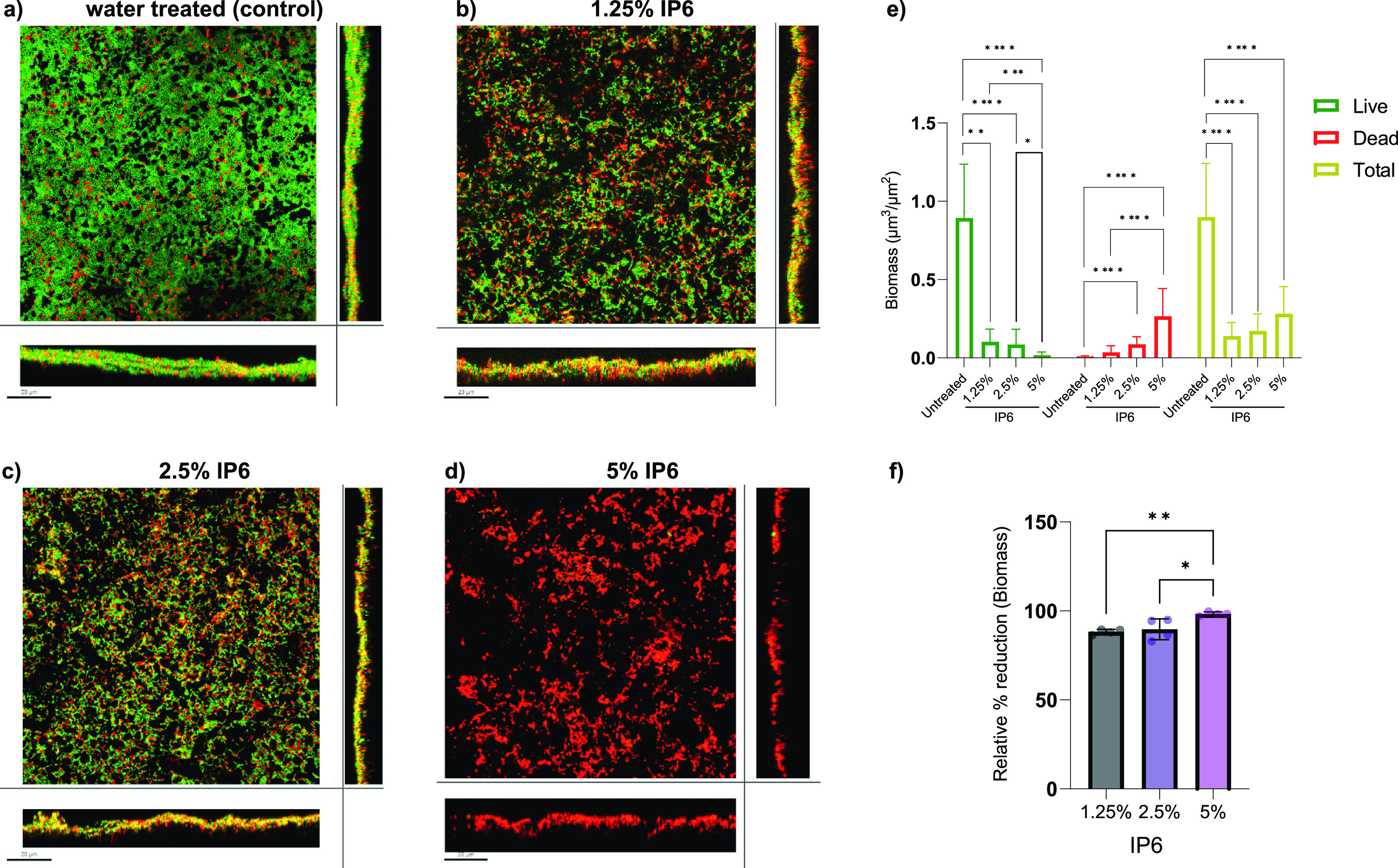
Confocal laser-scanning microscopy (CLSM) of live (green)/dead (red)-stained 72-h E. faecalis ATCC 29212 mono-species biofilms that were developed on HA coupons and treated with water (a) or 1.25% (b), 2.5% (c), or 5% (d) IP6 for 5 min. The square images show biofilm projection through the *x*-*y* plane, the bottom rectangle shows the *x*-*z* projection, and the right rectangle shows the *y*-*z* plane. Scale bar, 20 μm. (e) Biomass (green channel and red channel) of the mono-species biofilms treated with IP6 for 5 min. (f) Biomass (green channel) relative percent reduction (compared to untreated biofilms) of the mono-species biofilms. Data are expressed as means of four independent experiments. Error bars represent SD; *, *P* < 0.05; **, *P* < 0.01; ****, *P* < 0.0001.

**FIG 5:**
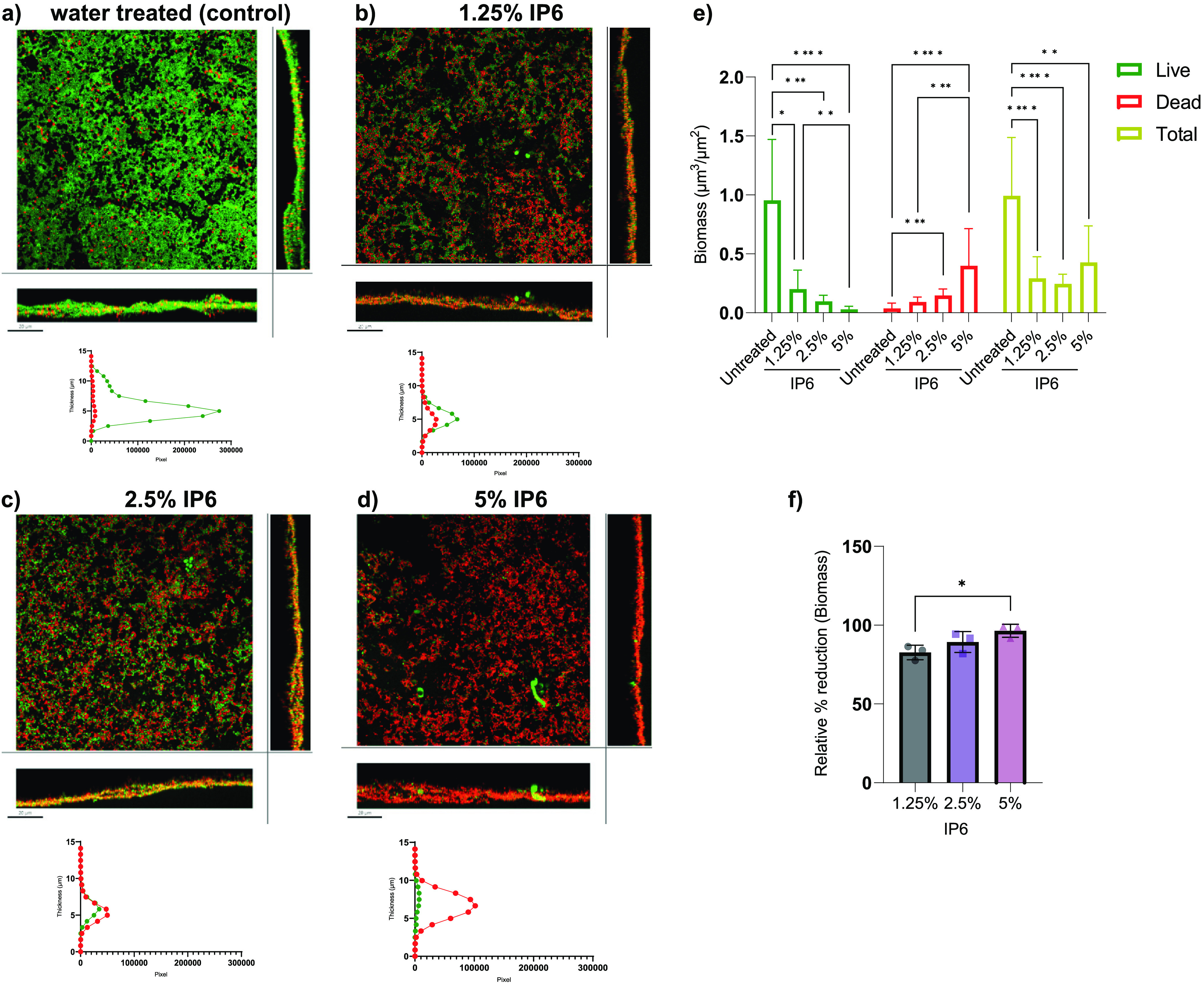
Confocal laser-scanning microscopy (CLSM) of live (green)/dead (red)-stained 72-h E. faecalis ATCC 29212 and C. albicans ATCC 90028 dual-species biofilms that were developed on HA coupons and treated with water (a) or 1.25% (b), 2.5% (c), or 5% (d) IP6 for 5 min. The square images show biofilm projection through the *x*-*y* plane, the bottom rectangle shows the *x*-*z* projection, and the right rectangle shows the *y*-*z* plane. Scale bar, 20 μm. The graphs below the CLSM images represent the distribution of pixel average of both stains along the biofilm thickness. Pixel average was calculated from three independent experiments. (e) Biomass (green channel and red channel) of the dual-species biofilms treated with IP6 for 5 min. (f) Biomass (green channel) relative percent reduction (compared to untreated biofilms) of the dual-species biofilms. Data are expressed as means of three independent experiments for CLSM and four independent experiments for CFU analysis. Error bars represent SD; *, *P* < 0.05; **, *P* < 0.01; ***, *P* < 0.001; ****, *P* < 0.0001.

To assess antibiofilm activity of IP6 on more clinically relevant surfaces, HA coupons were used for biofilm formation in a continuous flow of a diminishing nutrient medium content along with exposure to shear force. The effect of IP6 over a clinically relevant exposure time of 5 min was assessed based on recovered CFU and confocal laser-scanning microscopy (CLSM). For E. faecalis ATCC 29212 mono-species biofilms, IP6 at 1.25%, 2.5%, and 5% led to a significant reduction in log_10_ (CFU) values compared with untreated controls (Fig. 3a), with relative percent mean reductions of E. faecalis CFU (compared to the untreated control) of 96.79%, 98.98%, and 99.70%, respectively (Table S3 in the supplemental material). For dual-species biofilms, 1.25%, 2.5%, and 5% IP6 led to significant reductions in E. faecalis log_10_ (CFU) values (Fig. 3b), with relative percent mean reductions for E. faecalis CFU of 97.32%, 99.09%, and 99.33%, respectively (Table S4). The presence of both E. faecalis and C. albicans in biofilms did not affect IP6 efficacy against E. faecalis. However, in all cases of IP6 treatment (1.25%, 2.5%, and 5%), no reduction in C. albicans ATCC 90028 log_10_ (CFU) values occurred (Fig. 3b). Live biofilm biomass was significantly reduced with all IP6 treatments (Fig. 4e). The biomass of dead cells increased in treated groups but was only significant for biofilms treated with 2.5% and 5% IP6. Treatment with 5% IP6 led to the highest reduction in live biomass, which reached a mean of 98.31% and was significantly higher than 1.25% and 2.5% IP6 (Fig. 4f). The same pattern of significant live biomass reduction was seen with dual-species biofilms treated with 1.25%, 2.50%, or 5% IP6 (Fig. 5e). The relative reduction in live biomass of dual-species biofilms after IP6 treatment showed that 5% IP6 caused a mean reduction of 96.44% (Fig. 5f). Comparison between the three treatments demonstrated that 5% IP6 achieved a statistically higher reduction in live biomass of dual-species biofilms than the reduction caused by 1.25% IP6 (Fig. 5f). CLSM images and associated graphs of live/dead mono-species and dual-species biofilms are shown in Fig. 4a to d and 5a to d.

### Effect of preconditioning HA coupons with IP6 on biofilm formation.

**FIG 6:**
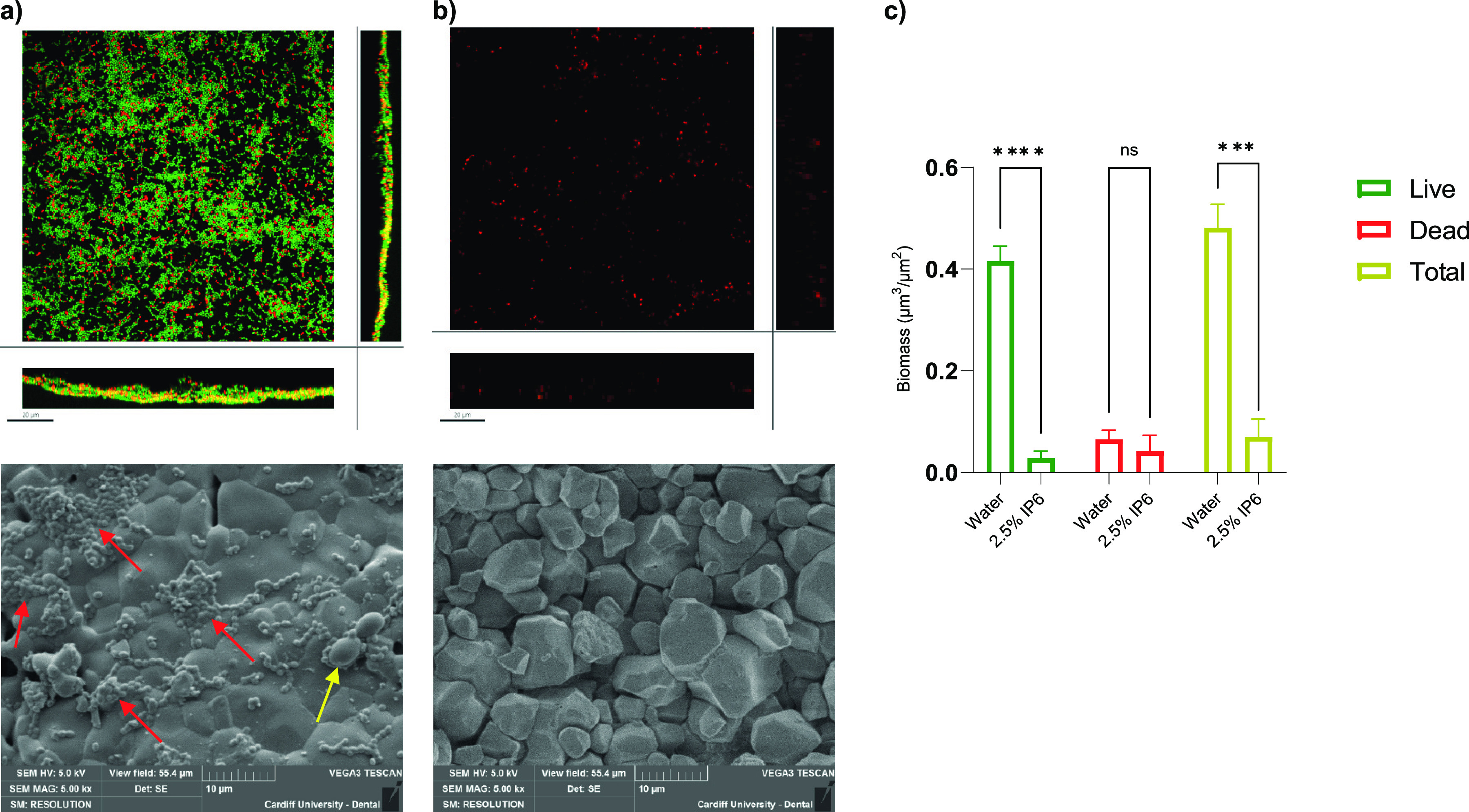
Dual-species biofilms of E. faecalis ATCC 29212 and C. albicans ATCC 90028 developed on HA coupons for 24 h that were either preconditioned with water (control) or 2.5% IP6. (a and b) Scanning electron microscopy (bottom) and confocal laser-scanning microscopy (top) images of live/dead-stained dual-species biofilms that were developed on HA coupons preconditioned with either water (a) or 2.5% IP6 (b) are shown. Red arrows indicate E. faecalis communities, while yellow arrows indicate C. albicans. (c) Graph bars represent the biomass of developed biofilms on HA coupons that were either preconditioned with water (control) or 2.5% IP6. Data are expressed as the means of three independent experiments. Error bars and values in parentheses represent SD; ****, *P* < 0.0001; ***, *P* < 0.001; ns, not significant.

The effect of preconditioning HA coupons with 2.5% IP6 on biofilm formation of dual-species biofilms of E. faecalis and C. albicans was investigated by CLSM and scanning electron microscopy (SEM). CLSM quantitative image analysis revealed significant reductions in total and live-cell biomass of biofilms formed on HA coupons preconditioned with 2.5% IP6 (*P* < 0.001 and *P* < 0.0001, respectively) (Fig. 6c). This was also evident in CLSM and SEM images (Fig. 6a and b). HA coupons preconditioned with water had a predominant number of live cells in the formed dual-species biofilm.

### Effect of IP6 on virulence gene expression and hyphal morphogenesis.

**FIG 7:**
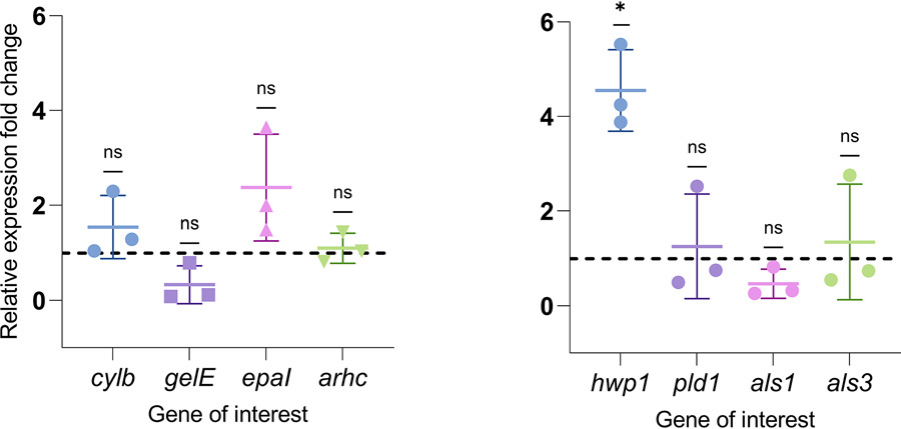
Effect of IP6 (0.16%) on E. faecalis ATCC 29212 (left) and C. albicans ATCC 90028 (right) gene expression determined by qPCR analysis. The analysis was performed using the 2^−ΔΔ^*^CT^* method relative to untreated control (dotted line value equal to 1). Data are expressed as means of three independent experiments. Error bars represent SD; *, *P* < 0.05; ns, not significant.

**FIG 8:**
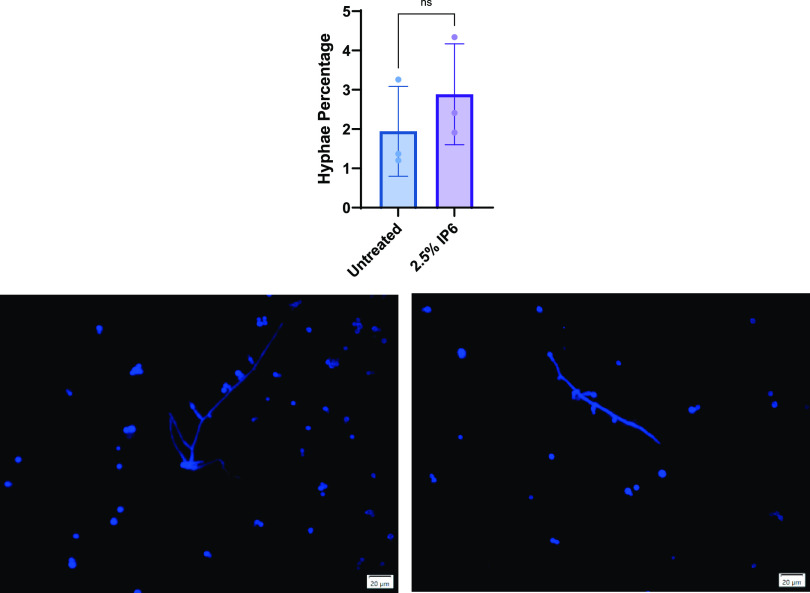
Candida albicans ATCC 90028 hyphal percentage in dual-species biofilm treated with 0.16% IP6 for 24 h compared to untreated (control) biofilm (top). Data are expressed as means of three independent experiments. Error bars represent SD; ns, not significant. Fluorescence images of a dual-species biofilm stained with calcofluor white were used to quantify C. albicans hyphae. The presence of C. albicans hyphae in untreated control biofilm (bottom left) and 0.16% IP6-treated biofilm (bottom right) is shown.

The relative expression of selected virulence genes of E. faecalis ATCC 29212 and C. albicans ATCC 90028 in biofilms following treatment with 0.16% IP6 was quantified. All analyzed E. faecalis genes demonstrated no significant changes in expression levels for IP6-treated biofilms compared to untreated biofilms. The expression of C. albicans genes was unchanged in treated biofilms with the exception of *hwp1* gene expression, which was upregulated (Fig. 7). To evaluate whether *hwp1* gene expression upregulation translated phenotypically, the percentage of hyphae after treatment with 0.16% IP6 was compared with that observed in untreated controls. The results showed that there was no significant change in hyphal development in treated biofilms (*P* = 0.40) (Fig. 8).

## DISCUSSION

IP6 is a natural compound with high biocompatibility that has been proposed as an alternative chelating agent to EDTA for root canal smear layer removal (29). Several studies have assessed the potential of IP6 for use in dentistry (35). However, its antimicrobial properties have not yet been adequately investigated. We previously examined the broad-spectrum antimicrobial activity of IP6 and showed clear activity against microorganisms (34). However, that work involved planktonic cultures and immature biofilms; hence, the antibiofilm activity of IP6 on mature robust biofilms, which better represent persistent endodontic infections, remained to be established. The findings from this study are the first detailing the antibiofilm activity of IP6 on the eradication of endodontic-related biofilms and its effectiveness in inhibiting biofilm formation when used as preconditioning agent on HA coupons.

Mono- and dual-species *in vitro* biofilms of E. faecalis and C. albicans were tested, as these species are frequently associated with persistent endodontic infection (36, 37). IP6 exhibited bactericidal activity against constituent cells of these mature biofilms, and concentrations that did not lead to complete eradication of biofilm cells led to reduced biofilm metabolic activity. It is worth highlighting that, in general, IP6 did not affect total biofilm biomass, even for concentrations where total bactericidal effects were reported, and this finding suggests that IP6 does not have “surfactant” effects on tested biofilms.

IP6 antibiofilm effects against 72-h mono and dual biofilms on HA coupons with a clinically relevant timing of 5 min were also determined. These biofilms were developed under sheer force with decreasing levels of nutrients in the medium. This arguably better represents the deprivation of nutrients that occurs as endodontic infections progress. IP6 displayed antibacterial effects on biofilms even over this short exposure time. Previous work revealed that IP6 is a potent chelating agent (29), a property attributed to its ability to chelate multivalent cations via its multiple negative charges (38, 39). Therefore, it might be hypothesized that IP6 antibacterial activity was through its high affinity for cations that are present in the bacterial cell envelope. It has also been suggested that antibacterial effects of chelating agents arise through disturbing essential metal metabolic processes (40). However, chelating agents have different activities and mechanisms of action based on their affinity for ions (41).

IP6’s antibiofilm activity could be through bactericidal activity against constituent cells and/or by acting as a biofilm matrix destabilizer by interacting with biofilm cations, which are known to play a role in biofilm stability by cross-linking the negatively charged polymers in a biofilm, thus reducing repulsion and promoting biofilm stability (42, 43). Collectively, the presented findings clearly show an effect of IP6 on mature biofilms. Although studies from the food industry have investigated IP6 antibacterial effects on planktonic bacteria, the antibiofilm activity and the associated mechanisms remain poorly researched. In the present study, IP6 had effects on bacterial cells, but no activity was evident against C. albicans. This observation might relate to the biocompatibility of IP6 toward eukaryotic cells (29) and could support the hypothetical antibacterial mechanism of action of IP6 given cell envelope differences between bacteria and fungi.

It should be noted that the inability of a compound to completely eradicate biofilms might not necessarily preclude its value in managing biofilm-associated infections. It is possible that even at sublethal concentrations, an impact on microbial fitness and modulation of virulence factors could arise (44, 45). Some studies have even shown that at sublethal concentrations, some antimicrobials can upregulate virulence gene expression (46). Therefore, an evaluation of targeted gene expression of E. faecalis and C. albicans dual-species biofilms following exposure to sublethal IP6 concentrations was investigated. Expression of tested genes was not affected by exposure to sublethal levels of IP6. The *als1* and *als3* C. albicans adhesin genes were also not altered by IP6 treatment. In contrast, expression of C. albicans *hwp1* was upregulated. However, associated changes in hyphal transformation were not detected.

Another notable observation was IP6’s ability to completely inhibit biofilm formation when used as a preconditioning agent. This property could be exploited in prevention strategies where root canal walls could be treated with IP6 to inhibit microbial adherence and biofilm formation. This would be important in preventing secondary infections due to microbial leakage from the oral cavity into the root canal system. This characteristic could also be exploited in conditioning obturation materials, or implants, to prevent microbial adherence to surfaces, thus increasing treatment success rates. It might be logical to assume that surface topographical changes induced by IP6 were instrumental in the occurrence of these effects. However, SEM images demonstrated that HA surfaces became more irregular and had more undulations following IP6 conditioning. This might contradict previous research where “rougher” surfaces typically were thought to increase surface area and offer protection from shear forces for bacterial adhesion and biofilm formation (47). A recent study reported the high adsorption of IP6 into HA, where it was absorbed as monolayer (48). It could be that IP6 affects HA surface wettability and resulting charge, possibly due to IP6’s negatively charged phosphate group. A negatively charged surface might reduce bacterial adhesion through repulsion given the negative net charge of bacteria. It is worth highlighting that there remains a lack of clarity regarding the effect of surface charge on bacterial adhesion (47), and some studies have shown that bacteria can overcome electrostatic repulsion through cation bridging (47, 49). More studies are needed to understand not only the observed antibacterial activity of IP6 on formed biofilms but also its ability to inhibit biofilms on different surfaces incorporating a wide range of microbial species.

In conclusion, the present study demonstrates for the first time the antibiofilm efficacy of IP6 against mature mono- and dual-species biofilms as well as its high-level biofilm formation inhibition activity when used as a surface conditioning agent. The findings of this study highlight the unique antibiofilm properties of IP6, which could be exploited in dental applications as well as other areas where biofilm inhibition and management are needed.

## MATERIALS AND METHODS

### Microorganisms and growth conditions.

Enterococcus faecalis ATCC 29212, an oral strain of E. faecalis (50), and Candida albicans ATTC 90028 were used to generate mature biofilms. Enterococcus faecalis was cultured on tryptone soya agar (TSA) and incubated aerobically at 37°C for 24 h. Candida albicans was cultured on Sabouraud dextrose agar (SDA) at 5% CO_2_ and 37°C for 48 h.

### Static biofilm formation.

Mono- and dual-species biofilms were developed on the well surfaces of 12-well plates that had been preconditioned with Dulbecco’s Modified Eagle’s Medium (DMEM) for 24 h. Briefly, microbial colonies were used to prepare suspensions in tryptone soya broth (TSB), which were then incubated at 37°C with agitation (125 rpm) in a shaker incubator for 3 to 4 h. For mono-species biofilms, bacterial suspensions were adjusted to a 0.5 McFarland standard using a DensiCHEK meter (bioMérieux). For the dual-species inoculum, a 1.0 McFarland standard of E. faecalis ATCC 29212 was mixed with an equal volume of a 1.0 McFarland standard of C. albicans ATTC 90028. One milliliter of microbial suspension was added to each well of the 12-well plate. The biofilm of the oral strain of E. faecalis was grown statically for 48 h, while biofilms of E. faecalis ATCC 29212 and dual biofilms were grown for 72 h. Biofilms were cultured aerobically at 37°C, and the medium was changed every 24 h.

### Effect of IP6 on biofilm metabolic activity and biomass.

After biofilm formation, spent medium was discarded, and biofilms were washed twice with phosphate-buffered saline (PBS). One milliliter of TSB and 1 mL of IP6 (Sigma), with a concentration range of 40% to 0.31% in water, were added to designated wells. Untreated biofilm and blank controls received 1 mL of TSB and 1 mL of sterile distilled water. The biofilms were incubated aerobically for 24 h at 37°C. After IP6 treatment, test solutions were removed, and the biofilms were washed twice with PBS. Two mL of 10% alamarBlue solution was added to each well, and the plates were incubated for 1 h at 37°C. Fluorescence of the wells was then measured using a microplate reader (Hidex, Finland). To assess total biomass, alamarBlue suspension was discarded, and a 2-mL volume of 0.1% crystal violet (CV) was added and incubated for 10 min at room temperature. The CV was removed, and the wells were washed twice with distilled water. Excess water was removed, and the plates were allowed to air dry for 30 min. Two milliliters of 70% ethanol was added to each well to elute bound CV, and the plate was incubated at room temperature for 10 min. The absorbance of the wells was then read using a microplate reader at 570 nm.

### Effect of IP6 on microbial regrowth.

After treatment of the biofilms with IP6 for 24 h, test solutions were removed from the wells, which were then washed twice with PBS. Two milliliters of sterile TSB was then added to all wells, followed by 18 to 24 h of aerobic incubation at 37°C. After incubation, regrowth was assessed by reading the absorbance of the wells with a microplate reader (Hidex, Finland) at 600 nm.

### Effect of IP6 on CFU reduction.

The IP6 concentrations that allowed bacterial regrowth were used to study the effects of IP6 on bacterial cell count reduction. After treatment of the biofilms with IP6 for 24 h, the solution was removed from the wells, which were then washed twice with PBS. The biofilms were then detached by scraping and vigorous pipetting, and subsequent enumeration of viable cells was performed.

### Biofilm formation on hydroxyapatite (HA) coupons in a CDC Biofilm Reactor and antibiofilm activity of IP6 on developed biofilms.

Center for Disease Control (CDC) Biofilm Reactors were assembled, and HA coupons were positioned in designated sites in the support rods before sterilization. The system flask was aseptically filled with 325 mL of 3 g/L TSB. For mono-species biofilms, the CDC Biofilm Reactor was inoculated with 1 mL of a 0.5 MacFarland standard E. faecalis ATCC 29212 bacterial suspension. One milliliter of a 0.5 MacFarland standard E. faecalis ATCC 29212 and 1 mL of a 0.5 MacFarland standard C. albicans ATTC 90028 were inoculated for dual-species biofilms. The CDC Biofilm Reactor was incubated as a batch system for 18 to 24 h at 37°C while placed over a stirrer plate for baffle stirring to produce shear stress. After incubation, the biofilm reactor were connected with a carboy containing sterile 1 g/L TSB to run a continuous flow system for an additional 48 h at a flow rate of 1.1 mL/min. The biofilms formed on HA coupons were then treated with three concentrations of IP6 (1.25%, 2.5%, and 5%) for 5 min, and the antibiofilm activity of IP6 was assessed as outlined below.

### Assessment of CFU from treated biofilms.

A single tube method (51) was used to assess the effect of IP6 on the number of recovered CFU from biofilms. Briefly, following biofilm development, the coupon-supporting rods were removed from the CDC Biofilm Reactor and washed using PBS. The HA coupons were removed and placed in conical tubes containing 6 mL of IP6 (1.2%, 2.5%, and 5%) or water. The conical tube had sterilized plastic splashguards to prevent droplets accessing areas where IP6 could not reach. After 5 min, HA coupons were washed in 35 mL of PBS, transferred to 6 mL of PBS, and exposed to two repeated cycles of vortex mixing (30 s) and water bath sonication (30 s), with a final vortex mix for 30 s. The recovered biofilm content was mixed by repeated pipetting 10 times. One milliliter of the mix was transferred to a sterile plastic tube containing 9 mL of sterile PBS. After vortex mixing at high speed for 10 s, CFU counts were measured.

### Confocal laser-scanning microscopy (CLSM) assessment of IP6-treated biofilms.

After biofilm development in the CDC Biofilm Reactor, the rods were washed and treated as described above. Biofilms were stained with live/dead BacLight bacterial viability mixture (Invitrogen, Ltd., UK) for 10 min at room temperature and immediately analyzed by CLSM. Five random fields of view were imaged for each coupon using an LSM880 Airyscan microscope (Zeiss, UK). Quantitative image analysis was performed using Comstat 2.1 (www.comstat.dk) to quantify live and dead biomass. CLSM micrographs were generated using Imaris viewer software (9.9.1 version).

### Effect of preconditioning HA coupons with IP6 on biofilm formation.

To assess the effect of preconditioning HA coupons with IP6 on biofilm formation, coupons were soaked in either 2.5% IP6 or sterile distilled water for 18 h under aseptic conditions. The rods were removed and then placed in an assembled sterile CDC Biofilm Reactor containing TSB. The CDC Biofilm Reactor was inoculated with 1 mL of a 0.5 McFarland standard level of both E. faecalis ATCC 29212 and C. albicans ATCC 90028 and incubated at 37°C under batch conditions and shear force for 18 to 24 h. Biofilm formation was assessed by CLSM and scanning electron microscopy (SEM). For CLSM, coupons were processed as described above, and for SEM, coupons were washed with PBS and placed immediately in 12-well plates containing 2.5% glutaraldehyde and incubated overnight. After incubation, the biofilms were dehydrated in 2 mL of an ethanol solution series (50%, 70%, 85%, 95%, and 100%) for 10 min at each concentration. Dehydration with 100% ethanol was repeated twice. The samples were then sputter coated with gold and imaged using a Tescan VAGA SEM system at 5 to 10 kV.

### Effect of IP6 on gene expression.

The effect of 0.16% IP6 on gene expression of 72-h-old dual-species biofilms developed in 12-well plates, as described above, was assessed. Developed biofilms were challenged with 0.16% IP6 for 24 h. The test solution was removed, and the wells were washed twice with PBS. One milliliter of RNAprotect bacteria reagent (Qiagen) was added and left at room temperature for at least 10 min. The biofilms were detached by scraping and vigorous pipetting. The resulting suspension was processed for RNA extraction using a Qiagen kit, and RNA was then reverse transcribed as per the manufacturer’s protocol (Qiagen). Quantitative PCR was performed using SYBR green mix (Bio-Rad) under the following thermal cycling conditions: 95°C for 2 min, 40 cycles of 95°C for 5 s, and 60°C for 20 s. Gene expression was normalized to the expression of housekeeping genes and the gene expression of treated biofilms was calculated relative to untreated biofilms (control) using the 2^−ΔΔ^*^Ct^* method. Target genes and primer sequences are provided in Tables S1 and S2 in the supplemental material.

### Effect of IP6 on C. albicans hyphal transformation.

To study the effect of IP6 on hyphal production, 72-h dual-species biofilms were prepared and treated with 0.16% IP6 as described above. After treatment, the medium was retrieved, and the biofilm was washed twice with PBS and fixed with 4% paraformaldehyde at room temperature for 1 h. The wells were washed with PBS and stained with 0.2% calcofluor white for 30 min at room temperature. Images were obtained using an Olympus IX53 fluorescence inverted microscope. The proportion of hyphae relative to total *Candida* fungal units (yeast and hyphae) was determined using ImageJ software version 1.48.

### Statistical analysis.

Statistical analysis was performed using GraphPad Prism 9.4.0. A parametric *t* test was used to compare differences between any two groups. A Kruskal-Wallis test or ordinary one-way analysis of variance (ANOVA) was used to compare more than two groups. A *P* value of less than 0.05 was considered significant. Relative fold change in gene expression analysis was performed using a one-sample *t* test compared to a hypothetical mean of 1.
