# Supplementary material for: Phytic Acid Demonstrates Rapid Antibiofilm Activity and Inhibits Biofilm Formation When Used as a Surface Conditioning Agent
Source: Microbiol Spectr. 2023 May 16;11(3):e00267-23. doi: 10.1128/spectrum.00267-23 (PMC10269681; doi:10.1128/spectrum.00267-23)
Supplement: Supplemental file 1 — Supplemental material. Download spectrum.00267-23-s0001.pdf, PDF file, 0.1 MB [file spectrum.00267-23-s0001.pdf]

Table S1 Primer sequences for *Enterococcus faecalis* virulence genes

**Target Gene**	**Primer sequence (5'-3')**	**Reference**
23S RNA<br>Housekeeping gene	F- CCTATCGGCCTCGGCTTAG<br>R-<br>AGCGAAAGACAGGTGAGAATCC	(1)
Gelatinase<br>(*gelE*)	F-CGGATTGGTTACACCATTATCC<br>R-TGCCACTCCTTATCCATTTTT	(2)
Cytolysin<br>(*cylb*)	F - GCTCTA ATTGACTCG GGGATT<br>R- CACTCTTGG AGCAATCGTGT	
ArgR family transcription factor (*ahrC*)	F - GTTGAACGTGTCGCCTTTTT<br>R -GCTTTTTCCTCGGATGATGA	
Enterococcal polysaccharide antigen<br>*(epal)*	F - CAAATTATCCCGAGCCAGAA<br>R – AGAATTGCTGAGCCGACTTC	

Table S2 Primer sequences for *Candida albicans* virulence genes (3)

**Target Gene**	**Primer sequence (5'-3')**
Actin 1 (*act 1*)<br>Housekeeping gene	F – TGCTGAACGTATGCAAAAGG<br>R – TGAACAATGGATGGACCAGA
Hyphal wall protein 1<br>(*hwp 1*)	F – TCTACTGCTCCAGCCACTGA<br>R – CCAGCAGGAATTGTTTCCAT
Agglutinin-like sequence 1<br>(*als 1*)	F - CCCAACTTGGAATGCTGTTT<br>R – TTTCAAAGCGTCGTTCACAG
Agglutinin-like sequence 3<br>(*als 3*)	F - CTGGACCACCAGGAAACACT<br>R – GGTGGAGCGGTGACAGTAGT
Phospholipase D<br>(*pld 1*)	F - GCCAAGAGAGCAAGGGTTAGCA<br>R – CGGATTCGTCATCCATTTCTCC

Table S3 Values of minimum, maximum AND standard deviation (SD) of relative percentage reduction of recovered CFU/ml from mono species biofilm of *E. faecalis* ATCC 29212 developed on HA coupons and treated with IP6 for 5 min

		**1.25%IP6**	**2.5% IP6**	**5% IP6**
**Relative% Reduction (CFU)**	Minimum	95.40	97.90	99.21
	Maximum	98.65	99.59	99.98
	Mean	96.79	98.98	99.70
	SD	1.37	0.75	0.35

Table S4 Values of minimum, maximum AND standard deviation (SD) of relative percentage reduction of recovered CFU/ml from dual species biofilm of *E. faecalis* ATCC 29212 and *C. albicans* ATCC 90028 developed on HA coupons and treated with IP6 for 5 min.

**Relative % reduction of *E. faecalis* ATCC 29212 CFU**				
**[IP6]**	**Mean**	**SD**	**Maximum**	**Minimum**
**1.25%**	97.32	2.86	99.51	93.12
**2.5%**	99.09	0.50	99.76	98.67
**5%**	99.33	0.69	99.90	98.43

References

1. Shepard BD, Gilmore MS. 2002. Differential expression of virulence-related genes in Enterococcus faecalis in response to biological cues in serum and urine. Infect Immun 70:4344-52.
2. Kim MA, Rosa V, Min KS. 2020. Characterization of Enterococcus faecalis in different culture conditions. Sci Rep 10:21867.
3. Morse DJ, Wilson MJ, Wei X, Bradshaw DJ, Lewis MAO, Williams DW. 2019. Modulation of Candida albicans virulence in in vitro biofilms by oral bacteria. Lett Appl Microbiol 68:337-343.
